# Mortality trends related to cardiogenic shock in heart failure patients aged 25 and older across the United States: A study utilizing the CDC WONDER database from 1999 to 2023

**DOI:** 10.1016/j.ijcha.2025.101732

**Published:** 2025-06-26

**Authors:** Abdullah Naveed Muhammad, Sivaram Neppala, Himaja Dutt Chigurupati, Muhammad Omer Rehan, Hamza Naveed, Rabia Iqbal, Bazil Azeem, Ahila Ali, Mushood Ahmed, Prakash Upreti, Mobeen Zaka Haider, Yasar Sattar, Jamal S. Rana, Gregg C. Fonarow

**Affiliations:** a Department of Cardiology, Dow Medical College, Dow University of Health Sciences, Karachi, Pakistan; b Division of Cardiology, The University of Texas Health Science Center, San Antonio, TX, USA; c Department of Internal Medicine, New York Medical College at Saint Michael’s Medical Center, Newark, NJ 07102, USA; d Department of Internal Medicine, Queen Elizabeth the Queen Mother Hospital, EKHUFT, Margate Kent, United Kingdom; e Department of Medicine, Rawalpindi Medical University, Rawalpindi, Pakistan; f Department of Cardiology, Sands-Constellation Heart Institute, Rochester Regional Health, Rochester, NY, USA; g Department of Cardiology, West Virginia University, Morgantown, WV, USA; h Department of Cardiology, The Permanente Medical Group, Oakland, CA, USA; i Division of Cardiology, University of California, Los Angeles, USA

**Keywords:** Cardiogenic shock, Heart failure, Age-adjusted mortality rates, Race, Ethnicity, Sex, Geographic regions

## Abstract

**Background:**

Cardiogenic shock (CS) remains crucial in mortality rates for heart failure (HF) patients. However, contemporary data on long-term mortality trends related to CS are limited. This study investigates demographic patterns and trends in CS mortality among HF patients over 25 years.

**Methods:**

Data from the Centers for Disease Control and Prevention’s Wide-ranging Online Data for Epidemiologic Research (CDC WONDER) database (1999–2023) included adults aged ≥25 diagnosed with HF and CS. Age-adjusted mortality rates (AAMRs) per 100,000 population and trends were analyzed using Joinpoint regression to find the average annual percent change (AAPC) and annual percent change (APC).

**Results:**

Between 1999 and 2023, there were 108,514 deaths linked to cardiogenic shock among heart failure patients, with AAMRs increasing from 1.2 to 4.6 per 100,000 (AAPC: 5.90). The most significant increases occurred from 2009 to 2021 (APC: 14.17), followed by a sustained rise from 2021 to 2023 (APC: 7.83). Men consistently exhibited higher AAMRs than women (2.4 vs. 1.3), and Black individuals had the highest mortality rates across all racial and ethnic groups. Furthermore, mortality rates were notably higher in rural areas compared to urban settings (1.7 vs. 1.5).

**Conclusion:**

In the past 25 years, CS-related mortality in HF patients has increased nearly fourfold. This trend highlights the need to investigate its causes, including potential deteriorating health outcomes or improved healthcare access. Special focus should be on high-risk groups like men, Black individuals, and rural residents, as targeted interventions could mitigate disparities and enhance outcomes.

## 1 Introduction

Cardiogenic shock (CS) is a critical condition defined by insufficient tissue perfusion caused by a marked decrease in cardiac output, usually due to severe ventricular dysfunction, resulting in end-organ hypoperfusion, hypoxia, and multi-organ failure [1]. Despite notable progress in diagnostic technologies, treatment methods, and critical care procedures, CS remains a substantial risk to patient survival, as 40–45 % of those affected perish within 30 days [[2], [3], [4]]. Although CS is fundamentally a clinical diagnosis, recent guidelines now establish precise criteria: (systolic blood pressure <90 mm Hg for ≥30 min, with or without mechanical support, Cardiac Index (CI) ≤2.2 L/min/m^2^ with support or <1.8 L/min/m^2^ without support, serum lactate levels > 2.0 mmol/L, and pulmonary wedge pressure of ≥ 15 mm Hg) [5,6].

While ischemic heart disease-related left ventricular dysfunction remains the leading cause of CS, it is crucial to recognize that chronic heart failure, arrhythmias, pericardial diseases, and valvular dysfunction also significantly contribute [7,8]. Over the last decade, we have witnessed a significant transformation in healthcare through the introduction of innovative therapies, including extracorporeal membrane oxygenation (ECMO), ventricular assist devices (VADs), and advancements in pharmacology. [9,10]. However, these developments have not led to universal improvements in patient outcomes, particularly among vulnerable groups.

Currently, more than 6.7 million adults in the United States are affected by heart failure, and this figure is projected to increase due to an aging population and higher survival rates from acute cardiovascular events [11]. In the context of heart failure, CS presents as a highly critical condition influenced by the combination of progressive ventricular dysfunction, systemic inflammation, and neurohormonal activation [12]. Prior research shows that mortality rates from CS are notably elevated in individuals with comorbid conditions like diabetes mellitus and chronic kidney disease, particularly among older adults. [13]. Additionally, disparities in race, gender, and socioeconomic status significantly influence patient outcomes. [1,14].

While the management of heart failure and CS has advanced with the adoption of new therapies like SGLT2 inhibitors and the increased use of mechanical circulatory support devices, critical questions remain regarding their overall effectiveness on population-level outcomes. Research has shown that interventions can improve survival rates among this patient population, although their efficacy has varied across different subsets [15]. Understanding these trends is crucial for identifying disparities and developing effective strategies to improve outcomes. This study examines the evolving trends and variations in outcomes related to cardiogenic shock-induced mortality among heart failure patients aged 25 and older in the United States.

## 2 Methods

### 2.1 Study design and cohort

This study employed the CDC WONDER Multiple Cause of Death database spanning the years 1999 to 2023, which comprises publicly accessible, de-identified mortality data from the United States, as derived from death certificates. Participants aged 25 years and older were included in the analysis if cardiogenic shock (ICD-10 code R57.0) and HF (I11.0, I13.0, I13.2, and I50. x) were indicated as an underlying or contributing cause of death. Records lacking essential demographic variables (age, sex, or race/ethnicity) were excluded from the study. The data were stratified based on age, sex, race/ethnicity, and U.S. Census region. Age-adjusted mortality rates were calculated using the direct method, per the 2000 U.S. standard population. Given that the dataset is publicly available and does not include identifiable information, Institutional Review Board (IRB) approval was deemed unnecessary. Nonetheless, the research rigorously adhered to the Strengthening the Reporting of Observational Studies in Epidemiology (STROBE) guidelines, ensuring a high standard of scientific integrity in the reliability of our findings.

### 2.2 Selection criteria

This study included:

- U.S. adults aged 25 and above.
- The state registries reported deaths between 2000 and 2023 with issued death certificates to the CDC-WONDER database.
- Deaths due to CS and HF.

This study excluded:

- Deaths not reported to state registries.

### 2.3 Data extraction

This study has created a detailed dataset that covers key mortality-related factors, including population size, year of death, geographic location, demographic characteristics, urban–rural classification, regional distribution, and state-specific categories. The demographic information includes key variables such as age and race/ethnicity. Death locations are categorized among various healthcare settings, including outpatient facilities, emergency rooms, and inpatient hospitals, as well as distinguishing cases where death occurred on arrival or with an unknown status, alongside deaths occurring at home, in hospice care, nursing homes, or long-term care facilities. Classifications of race and ethnicity consist of Hispanic, non-Hispanic (NH) White, NH Black, and NH Other. These carefully gathered parameters have been validated through analysis of the CDC WONDER database, enabling a detailed understanding of mortality trends and their implications.

To ensure an accurate population assessment, we employed the National Center for Health Statistics' Urban-Rural Classification Scheme, which categorizes counties as urban (including large central metropolitan, large fringe metropolitan, medium metropolitan, and small metropolitan areas) or nonmetropolitan (comprising micropolitan and noncore areas), based on the 2013 U.S. Census. Additionally, utilizing definitions from the U.S. Census Bureau, regions were delineated into the Northeast, Midwest, South, and West, thereby enhancing the specificity and relevance of our findings. Through these robust classifications, our study provides critical insights into mortality patterns across diverse populations.

### 2.4 Statistical analysis

The study thoroughly examined trends in nationwide mortality by analyzing the Age-Adjusted Mortality Rates (AAMR) per 100,000 individuals. This process involved precise calculations of total fatalities related to CS within the heart failure patient population for each analyzed year. Adhering to standard research methodologies, the AAMR was determined by standardizing CS-associated deaths against the 2000 U.S. population, generating 95 % confidence intervals for accuracy. To calculate the annual percent change (APC) and its related 95 % confidence interval for AAMR, we used the JoinPoint Regression Program (version 4.9.0.0, National Cancer Institute, Bethesda, MD, USA). By analyzing AAMRs, we facilitated meaningful comparisons of mortality rates across various populations and periods. The findings uncovered significant mortality trends, identifying notable fluctuations over time through log-linear regression models.

## 3 Results

**Fig. 1:**
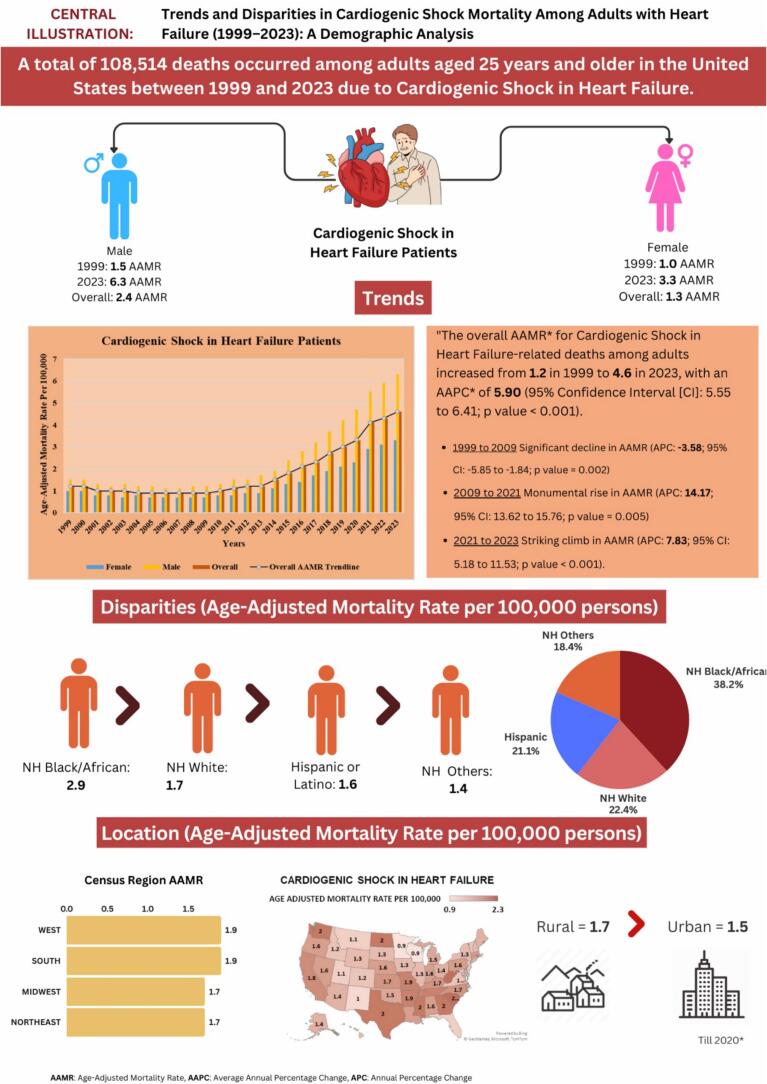
Central illustration depicting trends in demographics and disparities in cardiogenic shock-related mortality among heart failure patients in the United States from 1999 to 2023.

Between 1999 and 2023, Cardiogenic Shock among Heart Failure patients accounted for a total of 108,514 deaths among adults aged 25 + years in the United States **(**Supplementary Table 1**).** These fatalities were distributed across various settings, with the leading most occurring in medical facilities (90.3 %), 3.9 % in nursing homes/long-term care facilities, 3.4 % at the decedents’ homes, 1.7 % in hospice facilities, and 0.7 % at other locations **(**Supplementary Table 2**).** The central illustration summarizing the study's characteristics and findings is presented in Fig. 1**.**

### 3.1 Annual trends for cardiogenic shock in heart failure-related age-adjusted mortality rate (AAMR)

The age-adjusted mortality rate (AAMR) for CS in HF-related deaths among adults aged 25 and older has risen sharply from 1.2 per 100,000 in 1999 to 4.6 per 100,000 in 2023, demonstrating an Average Annual Percentage Change (AAPC) of 5.90 (95 % CI: 5.55 to 6.41; p < 0.001). Following a significant decline from 1999 to 2009 (APC: −3.58; 95 % CI: −5.85 to −1.84; p = 0.002), the AAMR experienced a substantial increase from 2009 to 2021 (APC: 14.17; 95 % CI: 13.62 to 15.76; p = 0.005199), continuing with another notable rise from 2021 to 2023 (APC: 7.83; 95 % CI: 5.18 to 11.53; p < 0.001) **(**Supplementary Table 3**).**

### 3.2 Cardiogenic shock in heart failure-related AAMR stratified by sex

Throughout the study period, it is evident that adult men have consistently shown significantly higher AAMRs compared to adult women, with men averaging an AAMR of 2.4 (95 % CI: 2.3–2.5) versus 1.3 for women (95 % CI: 1.3–1.4) over the whole study period from 1999 to 2023. Notably, both genders have experienced an upward trend in AAMRs from 1999 to 2023; however, the increase among men was notably more striking, with a reported AAPC of 6.31 (CI: 6.00 to 6.81) (p < 0.001) for men, compared to 5.28 (CI: 4.83 to 6.23) (p < 0.001) for women.

**Fig. 2:**
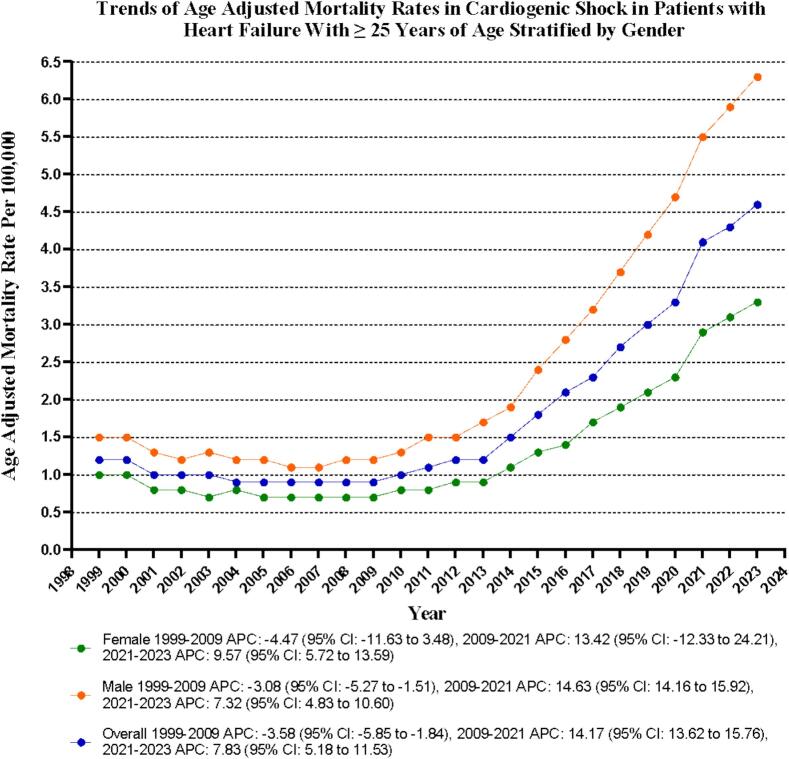
Overall and Sex-Stratified Cardiogenic shock-related age-adjusted mortality rates per 100,000 Adults with Heart Failure in the United States, 1999 to 2023.

For men, the AAMR decreased from 1.5 in 1999 to 1.2 in 2009 (APC: −3.08; 95 % CI: −5.27 to −1.51, p = 0.0004), followed by a dramatic surge to 5.5 by 2021 (APC: 14.63; 95 % CI: 14.16 to 15.92, p < 0.001) and a further notable rise to 6.3 by 2023 (APC: 7.32; 95 % CI: 4.83 to 10.60, p < 0.001). In comparison, adult women showed no significant change in AAMR from 1999 to 2021, and ultimately, a substantial rise to 3.3 by 2023 (APC: 9.57; 95 % CI: 5.72–13.59, p < 0.001). The wider confidence intervals for women from 2009 to 2021 likely reflect lower annual death counts and increased mortality variability during this period **(**Supplementary Table 4 **and** Fig. 2**).**

### 3.3 Cardiogenic shock in heart failure-related AAMR stratified by race/ethnicity

Significant differences in AAMRs exist among various racial and ethnic groups, with non-Hispanic (NH) Black individuals facing the highest rates. Specifically, the average AAMRs during the study period from 1999 to 2023 are as follows: NH Black: 2.9 (95 % CI: 2.7–3.2); NH White: 1.7 (95 % CI: 1.6–1.7); Hispanic: 1.6 (95 % CI: 1.5–1.8); and NH Other: 1.4 (95 % CI: 1.2–1.6).

**Fig. 3:**
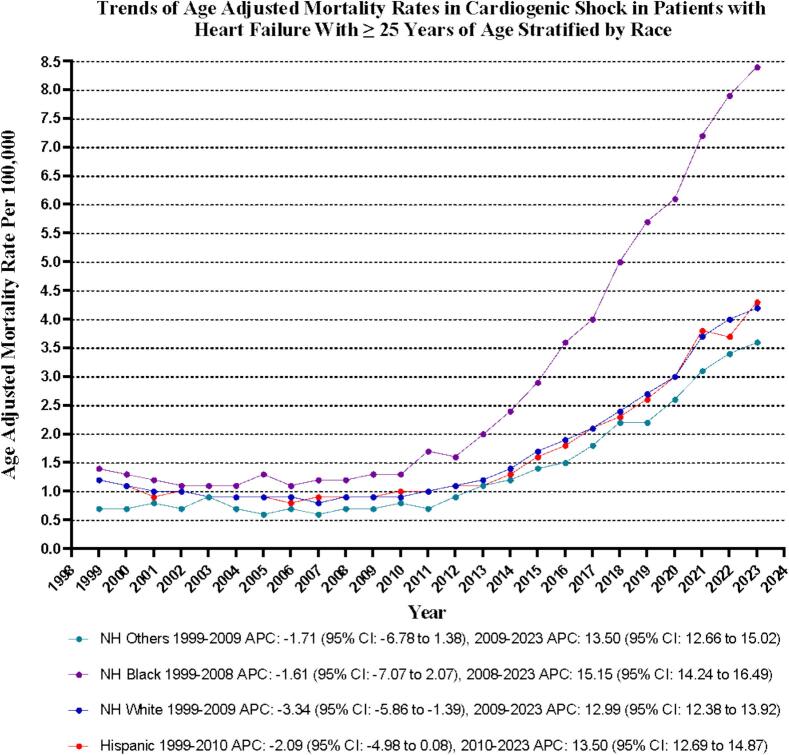
Cardiogenic shock-related age-adjusted mortality rates per 100,000 Stratified by Race in Adults with Heart Failure in the United States, 1999 to 2023.

Moreover, the data illustrate a dramatic increase in AAMRs across all racial groups from 1999 to 2023, with NH Blacks experiencing the most significant rise. The AAPC highlights this trend: NH Black: AAPC: 8.56 (95 % CI: 7.78 to 9.96; p < 0.001); NH Other: AAPC: 6.90 (95 % CI: 6.21 to 7.95; p < 0.001); Hispanic: AAPC: 6.07 (95 % CI: 5.44 to 6.74; p < 0.001); NH White: AAPC: 5.88 (95 % CI: 5.42 to 6.42; p < 0.001) **(**Supplementary Table 5 **and** Fig. 3**).**

### 3.4 Cardiogenic shock in heart failure-related AAMR stratified by geographical regions

Significant disparities in AAMRs are evident across states during the study period from 1999 to 2023, with rates as low as 0.9 (95 % CI: 0.8–0.9) in Wisconsin and as high as 2.1 (95 % CI: 2.0–2.3) in West Virginia. Alarmingly, states in the top 90th percentile—such as Georgia, Mississippi, North Dakota, Rhode Island, South Carolina, Texas, Washington, and West Virginia—exhibit AAMRs approximately 1.5 times greater than those in the bottom 10th percentile, which includes Colorado, Florida, Idaho, Maryland, Minnesota, Montana, New Jersey, New Mexico, Utah, Vermont, Virginia, and Wisconsin **(**Supplementary Table 6**).**

**Fig. 4:**
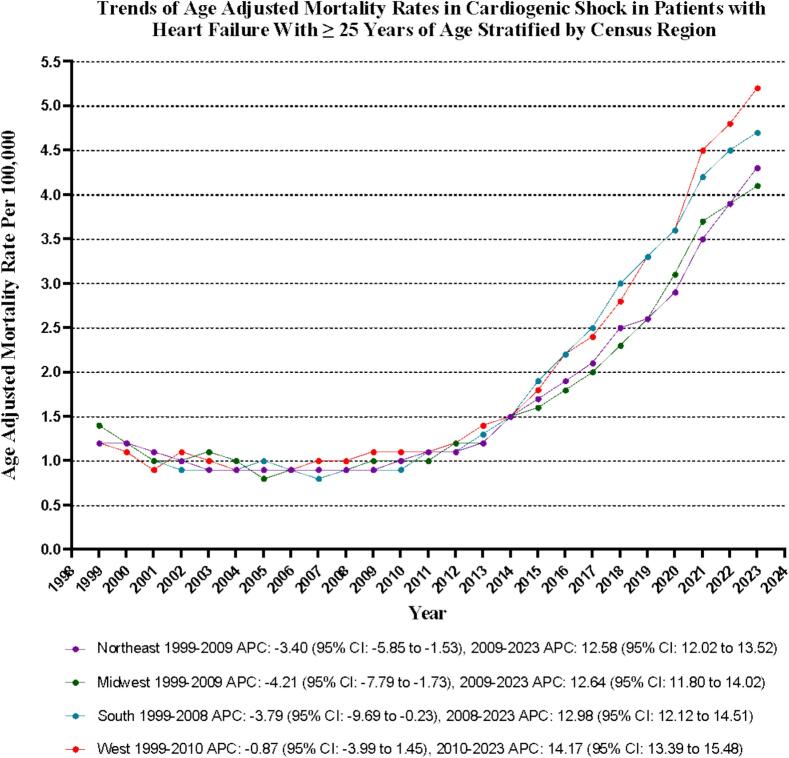
Cardiogenic shock-related age-adjusted mortality rates per 100,000 Stratified by Regions in Adults (≥25 Years) with Heart Failure in the United States, 1999 to 2023.

Analyzing data over the study period reveals that the Western and Southern regions have the highest mortality rates, each reporting an AAMR of 1.9 (95 % CI: 1.8–2.0 and 1.8–1.9, respectively). The Midwestern and Northeastern regions follow closely, with an AAMR of 1.7 (95 % CI: 1.6 to 1.8) **(**Supplementary Table 7 **and** Fig. 4**).**

**Fig. 5:**
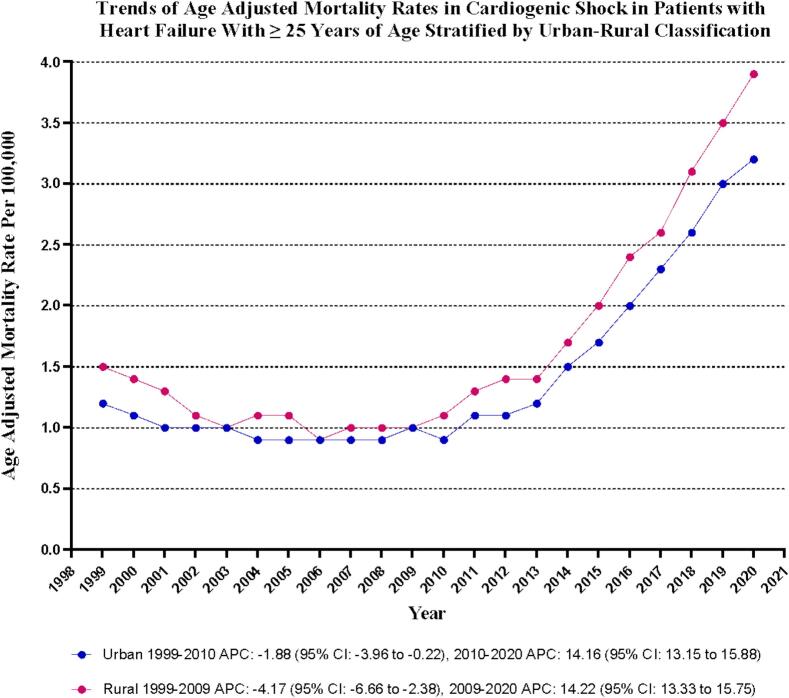
Cardiogenic shock-related age-adjusted mortality rates per 100,000 Stratified by Urbanization in Adults (≥25 Years) with Heart Failure in the United States, 1999 to 2023.

Throughout the study period, rural areas exhibited slightly higher AAMRs than urban areas, with overall AAMRs recorded at 1.7 (95 % CI: 1.7–1.8) for rural areas and 1.5 (95 % CI: 1.5–1.5) for urban areas. Notably, both urban and rural AAMRs increased significantly from 1999 to 2023, with a more notable rise in urban regions [Urban: AAPC: 5.45, (CI: 5.06 to 6.14) (p-value < 0.001); Rural: AAPC: 5.06, (CI: 4.67 to 5.73) (p-value < 0.001)] **(**Supplementary Table 8 **and** Fig. 5**).**

## 4 Discussion

Our thorough analysis of mortality data from the CDC WONDER, spanning from 1999 to 2023, highlights critical trends in Cardiogenic Shock (CS) mortality among individuals with Heart Failure (HF). The insights gained from our study are vital for effectively understanding and combating cardiogenic shock mortality, and they include the following key findings:

- Cardiogenic shock caused an alarming 108,514 fatalities in adults aged 25 and older with heart failure in the United States.
- From 2009 to 2021, the AAMR for HF-related CS increased significantly, reaching a peak APC of 14.17. This upward trend persisted into 2023, showing an APC of 7.83.
- Men face higher AAMRs than women, reflecting a dramatic increase from 2009 to 2023 for both genders.
- NH Black individuals have the highest AAMRs, with significant increases across all racial groups, particularly among NH Blacks.

Our analysis indicates that between 1999 and 2023, mortality related to CS in HF patients rose nearly threefold. While a decrease in mortality was noted from 1999 to 2009 (APC: −3.58), likely attributable to advancements in heart failure management- such as guideline-directed medical therapy (GDMT), early revascularization, and enhanced acute care [9,11]- the significant increase in annual average mortality rate (AAMR) from 2009 to 2021 (APC: 14.17) indicates that current management strategies may not adequately cope with the increasing complexity of HF phenotypes. This is particularly concerning as the population ages and comorbidities like diabetes and chronic kidney disease (CKD) escalate [13,16]. The persistent rise in mortality from 2021 to 2023 (APC: 7.83) might be linked to the COVID-19 pandemic, which hindered the management of chronic diseases and access to timely heart care. Delayed hospital visits decreased follow-up adherence, and overwhelmed healthcare systems probably worsened the outcomes [17,18].

In contrast to our findings, the Korean National Health Insurance database indicated a drop in overall CS mortality from 2010 to 2020. Nevertheless, the mortality specific to HF-CS remained elevated, aligning with our results [19]. The literature regarding CS in AMI patients presents contradictory views. Some research suggests a rise in CS incidence and a reduction in mortality, whereas other studies highlight ongoing in-hospital mortality despite the introduction of new treatments [[20], [21], [22]]. While advanced mechanical circulatory support (MCS) options, such as Impella and ECMO, have demonstrated efficacy in certain instances, their use is restricted due to cost, availability, and potential complications [9,15].

The increase in mortality after 2009 among adults with cardiogenic shock reflects several factors. First, better identification and documentation of cardiogenic shock—more frequent coding as a principal diagnosis—may have inflated reported mortality rates. Second, changes in ICD coding and hospital protocols around 2009 likely shifted the diagnostic categorization and case mix in national databases. Lastly, the increased use of advanced mechanical circulatory support—from intra-aortic balloon pumps to percutaneous ventricular assist devices—may have resulted in a concentration of higher-risk cases in recent years. This trend aligns with findings by Sterling et al., who, in their analysis of 9,789 patients with cardiogenic shock from 2009 to 2019, reported persistent in-hospital mortality rates (30.2 %) and increased use of mechanical support and ventilation over time [23].

Our analysis reveals notable gender disparities, with men displaying higher AAMRs compared to women, which underscores recognized sex differences in cardiovascular disease outcomes [24]. Despite a general decrease in cardiovascular mortality, significant disparities remain. Factors affecting women, such as cardiotoxic cancer treatments and cardiovascular stressors associated with pregnancy, may contribute to under-recognition and treatment delays [25]. Furthermore, the years following COVID-19 showed heightened mortality rates for both genders.

Our analysis revealed notable racial disparities, particularly with non-Hispanic Black individuals facing the highest AAMR. This aligns with prior research showing that systemic factors such as restricted access to advanced treatments (like ECMO and mLVAD), insurance disparities, and healthcare discrimination contribute to the issue [26,27]. Additionally, rural regions exhibited higher mortality rates compared to urban settings, indicating geographic challenges in accessing emergency cardiovascular care [15,28]. These results underscore the need to strengthen health systems and ensure equitable distribution of resources to reduce CS-related mortality.

While innovative therapies such as ARNIs, SGLT2 inhibitors, and device-based treatments have greatly enhanced outcomes for chronic heart failure, their advantages may not be fully realized in patients experiencing acute cardiogenic shock, which is often characterized by severe decompensation and multi-organ failure. Issues such as disparities in healthcare access, insufficient adoption of guideline-directed therapies, and the increasing prevalence of multimorbidity can further diminish the overall effectiveness of these interventions. Additionally, many clinical trials have either inadequately represented or completely excluded patients with cardiogenic shock, limiting the applicability of their findings to this vulnerable population. As a result, the observed increase in mortality may reflect persistent clinical and systemic challenges that continue despite advancements in care treatment [29,30].

### 4.1 Limitations

This study highlights several notable limitations, primarily due to its retrospective design. The reliance on death certificates obtained from the CDC WONDER database raises pertinent concerns regarding potential misdiagnoses, which may engender misclassification bias. Furthermore, the use of the 2000 U.S. standard population for age adjustment may limit comparability over a 25-year period, particularly given the demographic shifts that have occurred during this time. Additionally, the absence of laboratory data, medication histories, and clinical insights into overall health, comorbidities, and treatments hampers a comprehensive analysis of mortality trends. However, in contrast to existing literature, this research emphasizes explicitly adults aged 25 and older across diverse racial backgrounds, thereby furnishing an elaborate examination of mortality trends associated with cardiogenic shock within a heterogeneous cohort. Spanning the years 1999 to 2023, the findings provide a longitudinal perspective that enhances the understanding of the evolution of cardiogenic shock mortality over more than two decades. Moreover, the CDC WONDER database does not differentiate among heart failure phenotypes (e.g., HFrEF, HFpEF) nor does it classify the etiologies of cardiogenic shock, such as acute myocardial infarction, progressive heart failure, or mixed shock states (e.g., sepsis-associated cardiogenic shock), thereby constraining our capacity to interpret cause-specific trends. Future investigations utilizing clinical registries may yield more nuanced insights.

## 5 Conclusion

The analysis reveals significant demographic and geographic disparities in mortality rates, underscoring the need for immediate action. Notably, the alarming increase in mortality from cardiogenic shock among heart failure patients has risen from 1.2 per 100,000 in 1999 to 4.6 per 100,000 in 2023, showing an AAPC of 5.9. This trend particularly affects men, Black individuals, and individuals residing in both rural and urban areas, highlighting the need for urgent and targeted public health initiatives. Implementing effective strategies that address these inequities and ensure everyone has equal access to healthcare services is essential. Taking prompt action can significantly enhance health outcomes for this vulnerable group.

CRediT authorship contribution statement

ANM and SN led the study and contributed to the analysis and writing of the original manuscript. YS, JSR, and GCF supervised the project and helped with editing. The rest of the authors assisted in writing the manuscript.

Ethical considerations

This analysis utilized de-identified disease surveillance data from CDC databases; therefore, ethical approval was not required.

Funding support and author disclosures

The authors declare no relevant relationships related to the content of this paper.

## CRediT authorship contribution statement

**Abdullah Naveed Muhammad:** Visualization, Methodology, Formal analysis, Data curation. **Sivaram Neppala:** Writing – review & editing, Writing – original draft, Validation, Formal analysis. **Himaja Dutt Chigurupati:** Writing – original draft, Validation. **Muhammad Omer Rehan:** Validation, Formal analysis. **Hamza Naveed:** Validation, Methodology. **Rabia Iqbal:** Methodology, Data curation. **Bazil Azeem:** Writing – review & editing, Methodology, Formal analysis. **Ahila Ali:** Writing – original draft, Formal analysis. **Mushood Ahmed:** Methodology, Formal analysis. **Prakash Upreti:** Writing – original draft, Formal analysis. **Mobeen Zaka Haider:** Visualization, Methodology. **Yasar Sattar:** Writing – review & editing, Supervision. **Jamal S. Rana:** Writing – review & editing, Supervision. **Gregg C. Fonarow:** Writing – review & editing, Supervision.

## Declaration of competing interest

The authors declare that they have no known competing financial interests or personal relationships that could have appeared to influence the work reported in this paper.

## Data Availability

Data will be made available on request.
