# Supplementary material for: Mortality trends related to cardiogenic shock in heart failure patients aged 25 and older across the United States: A study utilizing the CDC WONDER database from 1999 to 2023
Source: Int J Cardiol Heart Vasc. 2025 Jun 26;59:101732. doi: 10.1016/j.ijcha.2025.101732 (PMC12268090; doi:10.1016/j.ijcha.2025.101732)
Supplement: Supplementary Data 1 [file mmc1.docx]

**Supplemental Table 1: Cardiogenic Shock related mortalities in Adults with Heart Failure, Stratified by Sex and Race in the United States, 1999 to 2023**

| **Deaths** | | | | | | | | |
| --- | --- | --- | --- | --- | --- | --- | --- | --- |
| **Year** | **Overall** | **Women** | **Men** | **NH White** | **NH Black** | **NH Others** | **Hispanic** | **Population** |
| **1999** | 2,178 | 1,135 | 1,043 | 1,839 | 204 | 33 | 99 | 180,408,769 |
| **2000** | 2,078 | 1,071 | 1,007 | 1,740 | 201 | 31 | 97 | 181,984,640 |
| **2001** | 1,841 | 919 | 922 | 1,539 | 179 | 41 | 79 | 184,305,128 |
| **2002** | 1,823 | 926 | 897 | 1,501 | 177 | 38 | 101 | 186,208,028 |
| **2003** | 1,781 | 856 | 925 | 1,458 | 181 | 52 | 87 | 188,090,429 |
| **2004** | 1,785 | 910 | 875 | 1,456 | 186 | 38 | 103 | 190,205,384 |
| **2005** | 1,801 | 860 | 941 | 1,434 | 216 | 42 | 107 | 192,551,384 |
| **2006** | 1,746 | 849 | 897 | 1,409 | 198 | 46 | 93 | 195,019,359 |
| **2007** | 1,772 | 880 | 892 | 1,390 | 219 | 41 | 122 | 197,403,777 |
| **2008** | 1,886 | 891 | 995 | 1,485 | 219 | 62 | 118 | 199,795,090 |
| **2009** | 2,019 | 918 | 1,101 | 1,558 | 249 | 64 | 142 | 202,107,016 |
| **2010** | 2,095 | 971 | 1,124 | 1,608 | 264 | 70 | 150 | 203,891,983 |
| **2011** | 2,388 | 1,047 | 1,341 | 1,824 | 338 | 62 | 162 | 206,592,936 |
| **2012** | 2,587 | 1,121 | 1,466 | 1,942 | 346 | 93 | 200 | 208,826,037 |
| **2013** | 2,906 | 1,249 | 1,657 | 2,138 | 435 | 113 | 213 | 211,085,314 |
| **2014** | 3,504 | 1,544 | 1,960 | 2,552 | 544 | 133 | 263 | 213,809,280 |
| **2015** | 4,243 | 1,734 | 2,509 | 3,049 | 666 | 172 | 335 | 216,553,817 |
| **2016** | 5,063 | 2,007 | 3,056 | 3,560 | 892 | 190 | 404 | 218,641,417 |
| **2017** | 5,842 | 2,328 | 3,514 | 4,044 | 1,009 | 254 | 516 | 221,447,331 |
| **2018** | 6,926 | 2,683 | 4,243 | 4,715 | 1,291 | 323 | 577 | 223,311,190 |
| **2019** | 7,974 | 3,114 | 4,860 | 5,424 | 1,521 | 328 | 677 | 224,981,167 |
| **2020** | 8,963 | 3,406 | 5,557 | 6,048 | 1,661 | 412 | 822 | 226,635,013 |
| **2021** | 10,762 | 4,176 | 6,586 | 7,175 | 1,958 | 473 | 1,052 | 228,238,412 |
| **2022** | 11,859 | 4,668 | 7,191 | 7,981 | 2,175 | 550 | 1,060 | 229,508,599 |
| **2023** | 12,692 | 4,954 | 7,738 | 8,455 | 2,316 | 590 | 1,211 | 229,508,599 |
| **Total** | **108,514** | **45,217** | **63,297** | **77,324** | **17,645** | **4,251** | **8,790** | **5,161,110,099** |

Annual Population and Death Counts by Year. Population estimates reflect age-standardized U.S. figures used for rate calculations, not actual census totals

**Supplemental Table 2: Cardiogenic Shock-related Mortality, Stratified by Place of Death, in Adults with Heart Failure in the United States, 1999 to 2023.**

| **Deaths** | | | | |
| --- | --- | --- | --- | --- |
| **Year** | **Medical Facility** | **Nursing Home/Long-term Care Facility** | **Hospice Facility** | **Home** |
| **1999** | 1,773 | 307 | - | 88 |
| **2000** | 1,678 | 269 | - | 111 |
| **2001** | 1,556 | 177 | - | 93 |
| **2002** | 1,501 | 207 | - | 98 |
| **2003** | 1,475 | 200 | - | 91 |
| **2004** | 1,484 | 172 | - | 96 |
| **2005** | 1,518 | 166 | - | 97 |
| **2006** | 1,453 | 170 | - | 102 |
| **2007** | 1,501 | 142 | 10 | 95 |
| **2008** | 1,587 | 158 | - | 85 |
| **2009** | 1,633 | 162 | 21 | 91 |
| **2010** | 1,795 | 132 | 43 | 104 |
| **2011** | 2,059 | 144 | 45 | 116 |
| **2012** | 2,241 | 129 | 60 | 128 |
| **2013** | 2,581 | 134 | 53 | 112 |
| **2014** | 3,170 | 133 | 41 | 130 |
| **2015** | 3,834 | 141 | 96 | 151 |
| **2016** | 4,642 | 129 | 79 | 177 |
| **2017** | 5,419 | 134 | 112 | 154 |
| **2018** | 6,466 | 134 | 133 | 158 |
| **2019** | 7,373 | 146 | 192 | 221 |
| **2020** | 8,225 | 208 | 178 | 286 |
| **2021** | 10,014 | 151 | 243 | 284 |
| **2022** | 11,094 | 183 | 228 | 292 |
| **2023** | 11,925 | 157 | 256 | 281 |
| **Total** | **97,997** | **4,185** | **1,790** | **3,641** |

**Supplemental Table 3: Annual percent change (APC) of Cardiogenic Shock-related Age-Adjusted Mortality Rates per 100,000 in Adults with Heart Failure in the United States, 1999 to 2023**

| **Year Interval** | **APC (95% CI)** | **P-value** |  |
| --- | --- | --- | --- |
| **Overall** | | |  |
| 1999-2009 | -3.58* (-5.85 to -1.84) | 0.0024 |  |
| 2009-2021 | 14.17* (13.62 to 15.76) | 0.005199 |  |
| 2021-2023 | 7.83* (5.18 to 11.53) | < 0.001 |  |
| **Men** | | |  |
| 1999-2009 | -3.08* (-5.27 to -1.51) | 0.0004 |  |
| 2009-2021 | 14.63* (14.16 to 15.92) | < 0.001 |  |
| 2021-2023 | 7.32* (4.83 to 10.60) | < 0.001 |  |
| **Women** | | |  |
| 1999-2009 | -4.47 (-11.63 to 3.48) | 0.082783 |  |
| 2009-2021 | 13.42 (-12.33 to 24.21) | 0.316337 |  |
| 2021-2023 | 9.57* (5.72 to 13.59) | < 0.001 |  |
| **NH White** | | |  |
| 1999-2010 | -2.47* (-4.82 to -0.66) | 0.026395 |  |
| 2010-2021 | 14.27* (1.17 to 19.40) | 0.041192 |  |
| 2021-2023 | 8.42* (5.12 to 13.15) | < 0.001 |  |
| **NH Black** | | |  |
| 1999-2010 | 0.03 (-3.67 to 2.61) | 0.987003 |  |
| 2010-2019 | 18.75* (17.12 to 28.18) | 0.005599 |  |
| 2019-2023 | 10.06* (4.82 to 13.66) | 0.015197 |  |
| **NH Others** | | |  |
| 1999-2010 | -0.59 (-9.73 to 14.77) | 0.660268 |  |
| 2010-2021 | 14.31 (-10.74 to 24.92) | 0.282344 |  |
| 2021-2023 | 9.70* (3.94 to 14.95) | 0.002 |  |
| **Hispanic** | | |  |
| 1999-2006 | -4.74* (-12.40 to -1.79) | 0.009198 |  |
| 2006-2013 | 4.97* (0.07 to 11.70) | 0.04799 |  |
| 2013-2021 | 15.44* (14.35 to 20.40) | 0.0016 |  |
| 2021-2023 | 8.24* (5.08 to 12.38) | < 0.001 |  |
| **Rural areas** | | |  |
| 1999-2003 | -8.44* (-15.62 to -4.20) | 0.0008 |  |
| 2003-2009 | -2.00 (-4.97 to 19.20) | 0.866227 |  |
| 2009-2020 | 13.87* (12.52 to 15.81) | < 0.001 |  |
| **Urban areas** | | |  |
| 1999-2006 | -4.18* (-8.18 to -2.11) | 0.016397 |  |
| 2006-2012 | 4.09 (-1.00 to 9.72) | 0.075185 |  |
| 2012-2018 | 16.04* (12.08 to 20.60) | 0.0004 |  |
| 2018-2020 | 10.24* (7.50 to 13.91) | < 0.001 |  |
| **Northeast region** | | |  |
| 1999-2009 | -3.40* (-5.85 to -1.53) | 0.0004 |  |
| 2009-2023 | 12.58* (12.02 to 13.52) | < 0.001 |  |
| **South region** | | |  |
| 1999-2002 | -9.16* (-16.51 to -3.05) | 0.007199 |  |
| 2002-2010 | -0.90 (-3.52 to 12.55) | 0.824235 |  |
| 2010-2018 | 16.49 (-5.84 to 23.40) | 0.143571 |  |
| 2018-2021 | 12.37* (11.29 to 17.86) | < 0.001 |  |
| 2021-2023 | 6.50* (4.12 to 9.58) | < 0.001 |  |
| **Midwest region** | | |  |
| 1999-2005 | -6.90* (-12.82 to -4.26) | 0.013997 |  |
| 2005-2011 | 2.96 (-4.61 to 8.11) | 0.323935 |  |
| 2011-2018 | 11.84* (6.72 to 13.77) | 0.003199 |  |
| 2018-2021 | 17.84* (15.22 to 20.00) | < 0.001 |  |
| 2021-2023 | 5.61* (3.10 to 8.58) | < 0.001 |  |
| **West region** | | |  |
| 1999-2005 | -4.10* (-11.59 to -0.94) | 0.009998 |  |
| 2005-2012 | 3.84* (0.67 to 11.74) | 0.015597 |  |
| 2012-2021 | 15.80* (15.17 to 17.89) | < 0.001 |  |
| 2021-2023 | 8.95* (6.62 to 11.97) | < 0.001 |  |
| APC = annual percent change; NH = non-Hispanic; * Indicates that the annual percentage change (APC) is significantly different from zero at α = 0.05. AAMR = age-adjusted mortality rate. The data for urbanization is only available till 2020 in the CDC Wonder Database. | | |  |
|  |  |  |  |

**Supplemental Table 4: Overall and Sex‐Stratified Cardiogenic Shock-related Age-Adjusted Mortality Rates per 100,000 in Adults with Heart Failure in the United States, 1999 to 2023.**

| **Age-Adjusted Rate (95% CI)** | | | |
| --- | --- | --- | --- |
| **Year** | **Men** | **Women** | **Overall** |
| **1999** | 1.5 (1.4 - 1.6) | 1.0 (1.0 - 1.1) | 1.2 (1.2 - 1.3) |
| **2000** | 1.5 (1.4 - 1.6) | 1.0 (0.9 - 1.0) | 1.2 (1.1 - 1.2) |
| **2001** | 1.3 (1.2 - 1.4) | 0.8 (0.8 - 0.9) | 1.0 (1.0 - 1.1) |
| **2002** | 1.2 (1.1 - 1.3) | 0.8 (0.7 - 0.9) | 1.0 (0.9 - 1.0) |
| **2003** | 1.3 (1.2 - 1.4) | 0.7 (0.7 - 0.8) | 1.0 (0.9 - 1.0) |
| **2004** | 1.2 (1.1 - 1.2) | 0.8 (0.8 - 0.9) | 0.9 (0.9 - 1.0) |
| **2005** | 1.2 (1.1 - 1.3) | 0.7 (0.7 - 0.8) | 0.9 (0.9 - 1.0) |
| **2006** | 1.1 (1.0 - 1.2) | 0.7 (0.7 - 0.7) | 0.9 (0.9 - 0.9) |
| **2007** | 1.1 (1.0 - 1.1) | 0.7 (0.7 - 0.8) | 0.9 (0.8 - 0.9) |
| **2008** | 1.2 (1.1 - 1.3) | 0.7 (0.7 - 0.8) | 0.9 (0.9 - 0.9) |
| **2009** | 1.2 (1.2 - 1.3) | 0.7 (0.7 - 0.8) | 0.9 (0.9 - 1.0) |
| **2010** | 1.3 (1.2 - 1.4) | 0.8 (0.7 - 0.8) | 1.0 (0.9 - 1.0) |
| **2011** | 1.5 (1.4 - 1.6) | 0.8 (0.8 - 0.9) | 1.1 (1.0 - 1.1) |
| **2012** | 1.5 (1.5 - 1.6) | 0.9 (0.8 - 0.9) | 1.2 (1.1 - 1.2) |
| **2013** | 1.7 (1.6 - 1.8) | 0.9 (0.9 - 1.0) | 1.2 (1.2 - 1.3) |
| **2014** | 1.9 (1.8 - 2.0) | 1.1 (1.1 - 1.2) | 1.5 (1.5 - 1.6) |
| **2015** | 2.4 (2.3 - 2.5) | 1.3 (1.2 - 1.3) | 1.8 (1.7 - 1.8) |
| **2016** | 2.8 (2.7 - 2.9) | 1.4 (1.4 - 1.5) | 2.1 (2.0 - 2.1) |
| **2017** | 3.2 (3.1 - 3.3) | 1.7 (1.6 - 1.7) | 2.3 (2.3 - 2.4) |
| **2018** | 3.7 (3.6 - 3.8) | 1.9 (1.8 - 1.9) | 2.7 (2.6 - 2.7) |
| **2019** | 4.2 (4.1 - 4.3) | 2.1 (2.1 - 2.2) | 3.0 (3.0 - 3.1) |
| **2020** | 4.7 (4.5 - 4.8) | 2.3 (2.2 - 2.4) | 3.3 (3.3 - 3.4) |
| **2021** | 5.5 (5.4 - 5.7) | 2.9 (2.8 - 3.0) | 4.1 (4.0 - 4.1) |
| **2022** | 5.9 (5.7 - 6.0) | 3.1 (3.0 - 3.2) | 4.3 (4.3 - 4.4) |
| **2023** | 6.3 (6.2 - 6.5) | 3.3 (3.2 - 3.4) | 4.6 (4.6 - 4.7) |
| **Total** | **2.4 (2.3 - 2.5)** | **1.3 (1.3 - 1.4)** | **1.8 (1.8 - 1.8)** |

**Supplemental Table 5: Race‐Stratified Cardiogenic Shock-related Age-Adjusted Mortality Rates per 100,000 in Adults with Heart Failure in the United States, 1999 to 2023.**

| **Age-Adjusted Rate (95% CI)** | | | | |
| --- | --- | --- | --- | --- |
| **Year** | **NH White** | **NH Black** | **NH Others** | **Hispanic** |
| **1999** | 1.2 (1.2 - 1.3) | 1.4 (1.2 - 1.6) | 0.7 (0.5 - 1.0) | 1.2 (1.0 - 1.5) |
| **2000** | 1.1 (1.1 - 1.2) | 1.3 (1.2 - 1.5) | 0.7 (0.5 - 1.0) | 1.1 (0.9 - 1.4) |
| **2001** | 1.0 (0.9 - 1.0) | 1.2 (1.0 - 1.4) | 0.8 (0.6 - 1.1) | 0.9 (0.7 - 1.1) |
| **2002** | 1.0 (0.9 - 1.0) | 1.1 (1.0 - 1.3) | 0.7 (0.5 - 1.0) | 1.0 (0.8 - 1.3) |
| **2003** | 0.9 (0.9 - 1.0) | 1.1 (1.0 - 1.3) | 0.9 (0.7 - 1.2) | 0.9 (0.7 - 1.1) |
| **2004** | 0.9 (0.9 - 1.0) | 1.1 (0.9 - 1.3) | 0.7 (0.5 - 0.9) | 0.9 (0.8 - 1.1) |
| **2005** | 0.9 (0.9 - 1.0) | 1.3 (1.1 - 1.5) | 0.6 (0.4 - 0.8) | 0.9 (0.7 - 1.1) |
| **2006** | 0.9 (0.8 - 0.9) | 1.1 (1.0 - 1.3) | 0.7 (0.5 - 0.9) | 0.8 (0.6 - 1.0) |
| **2007** | 0.8 (0.8 - 0.9) | 1.2 (1.1 - 1.4) | 0.6 (0.4 - 0.8) | 0.9 (0.7 - 1.1) |
| **2008** | 0.9 (0.8 - 0.9) | 1.2 (1.0 - 1.3) | 0.7 (0.6 - 1.0) | 0.9 (0.7 - 1.0) |
| **2009** | 0.9 (0.9 - 1.0) | 1.3 (1.2 - 1.5) | 0.7 (0.6 - 1.0) | 0.9 (0.8 - 1.1) |
| **2010** | 0.9 (0.9 - 1.0) | 1.3 (1.2 - 1.5) | 0.8 (0.6 - 1.1) | 1.0 (0.9 - 1.2) |
| **2011** | 1.0 (1.0 - 1.1) | 1.7 (1.5 - 1.9) | 0.7 (0.5 - 0.9) | 1.0 (0.8 - 1.1) |
| **2012** | 1.1 (1.0 - 1.1) | 1.6 (1.4 - 1.8) | 0.9 (0.7 - 1.1) | 1.1 (1.0 - 1.3) |
| **2013** | 1.2 (1.1 - 1.2) | 2.0 (1.8 - 2.2) | 1.1 (0.9 - 1.3) | 1.1 (1.0 - 1.3) |
| **2014** | 1.4 (1.3 - 1.4) | 2.4 (2.2 - 2.6) | 1.2 (1.0 - 1.4) | 1.3 (1.1 - 1.5) |
| **2015** | 1.7 (1.6 - 1.7) | 2.9 (2.6 - 3.1) | 1.4 (1.2 - 1.6) | 1.6 (1.5 - 1.8) |
| **2016** | 1.9 (1.8 - 2.0) | 3.6 (3.4 - 3.9) | 1.5 (1.3 - 1.7) | 1.8 (1.6 - 1.9) |
| **2017** | 2.1 (2.1 - 2.2) | 4.0 (3.8 - 4.3) | 1.8 (1.6 - 2.0) | 2.1 (2.0 - 2.3) |
| **2018** | 2.4 (2.3 - 2.5) | 5.0 (4.7 - 5.3) | 2.2 (2.0 - 2.5) | 2.3 (2.1 - 2.5) |
| **2019** | 2.7 (2.7 - 2.8) | 5.7 (5.4 - 6.0) | 2.2 (2.0 - 2.5) | 2.6 (2.4 - 2.8) |
| **2020** | 3.0 (3.0 - 3.1) | 6.1 (5.8 - 6.4) | 2.6 (2.4 - 2.9) | 3.0 (2.8 - 3.3) |
| **2021** | 3.7 (3.6 - 3.8) | 7.2 (6.9 - 7.6) | 3.1 (2.8 - 3.3) | 3.8 (3.6 - 4.0) |
| **2022** | 4.0 (3.9 - 4.0) | 7.9 (7.5 - 8.2) | 3.4 (3.1 - 3.7) | 3.7 (3.5 - 3.9) |
| **2023** | 4.2 (4.1 - 4.3) | 8.4 (8.1 - 8.8) | 3.6 (3.3 - 3.9) | 4.3 (4.0 - 4.5) |
| **Total** | **1.7 (1.6 - 1.7)** | **2.9 (2.7 - 3.2)** | **1.4 (1.2 - 1.6)** | **1.6 (1.5 - 1.8)** |
| NH = non-Hispanic | | | | |
|  | | | | |

**Supplemental Table 6: Cardiogenic Shock related Age-Adjusted Mortality Rates per 100,000, Stratified by States, in Adults with Heart Failure in the United States, 1999 to 2023**

| **State** | **Age-Adjusted Rate (95% CI)** |
| --- | --- |
| Alabama | 1.6 (1.5 - 1.6) |
| Alaska | 1.4 (1.1 - 1.7) |
| Arizona | 1.4 (1.4 - 1.5) |
| Arkansas | 1.9 (1.7 - 2.0) |
| California | 1.8 (1.7 - 1.8) |
| Colorado | 1.2 (1.1 - 1.3) |
| Connecticut | 1.6 (1.5 - 1.7) |
| Delaware | 1.4 (1.2 - 1.6) |
| District of Columbia | 2.3 (1.9 - 2.6) |
| Florida | 1.1 (1.0 - 1.1) |
| Georgia | 2.0 (1.9 - 2.0) |
| Hawaii | 1.3 (1.1 - 1.4) |
| Idaho | 1.2 (1.1 - 1.4) |
| Illinois | 1.3 (1.3 - 1.4) |
| Indiana | 1.6 (1.6 - 1.7) |
| Iowa | 1.3 (1.2 - 1.4) |
| Kansas | 1.7 (1.6 - 1.8) |
| Kentucky | 1.7 (1.6 - 1.8) |
| Louisiana | 1.6 (1.5 - 1.7) |
| Maine | 1.4 (1.2 - 1.5) |
| Maryland | 1.2 (1.1 - 1.3) |
| Massachusetts | 1.6 (1.5 - 1.7) |
| Michigan | 1.5 (1.5 - 1.6) |
| Minnesota | 0.9 (0.9 - 1.0) |
| Mississippi | 2.0 (1.8 - 2.1) |
| Missouri | 1.9 (1.8 - 2.0) |
| Montana | 1.1 (1.0 - 1.3) |
| Nebraska | 1.6 (1.4 - 1.7) |
| Nevada | 1.6 (1.5 - 1.8) |
| New Hampshire | 1.3 (1.1 - 1.4) |
| New Jersey | 1.2 (1.1 - 1.2) |
| New Mexico | 1.0 (0.9 - 1.1) |
| New York | 1.3 (1.2 - 1.3) |
| North Carolina | 1.7 (1.6 - 1.8) |
| North Dakota | 2.0 (1.8 - 2.3) |
| Ohio | 1.4 (1.4 - 1.5) |
| Oklahoma | 1.5 (1.4 - 1.6) |
| Oregon | 1.6 (1.5 - 1.7) |
| Pennsylvania | 1.6 (1.6 - 1.7) |
| Rhode Island | 2.1 (1.9 - 2.3) |
| South Carolina | 2.1 (2.0 - 2.2) |
| South Dakota | 1.3 (1.1 - 1.5) |
| Tennessee | 1.9 (1.9 - 2.0) |
| Texas | 2.0 (1.9 - 2.0) |
| Utah | 1.1 (1.0 - 1.2) |
| Vermont | 1.2 (1.0 - 1.4) |
| Virginia | 1.2 (1.1 - 1.2) |
| Washington | 2.0 (2.0 - 2.1) |
| West Virginia | 2.1 (2.0 - 2.3) |
| Wisconsin | 0.9 (0.8 - 0.9) |
| Wyoming | 1.3 (1.1 - 1.6) |

**Supplemental Table 7: Cardiogenic Shock related Age-Adjusted Mortality Rates per 100,000, Stratified by Census Region, in Adults with Heart Failure in the United States, 1999 to 2023**

|  | **Census Region: NorthEast** | **Census Region: Midwest** | **Census Region:  South** | **Census Region:  West** |
| --- | --- | --- | --- | --- |
| **Year** | **Age-Adjusted Rate (95% CI)** | **Age-Adjusted Rate (95% CI)** | **Age-Adjusted Rate (95% CI)** | **Age-Adjusted Rate (95% CI)** |
| **1999** | 1.2 (1.1 - 1.3) | 1.4 (1.3 - 1.5) | 1.2 (1.1 - 1.3) | 1.2 (1.1 - 1.3) |
| **2000** | 1.2 (1.1 - 1.3) | 1.2 (1.1 - 1.3) | 1.2 (1.1 - 1.2) | 1.1 (1.0 - 1.2) |
| **2001** | 1.1 (1.0 - 1.2) | 1.0 (1.0 - 1.1) | 1.0 (0.9 - 1.1) | 0.9 (0.8 - 1.0) |
| **2002** | 1.0 (0.9 - 1.1) | 1.0 (0.9 - 1.1) | 0.9 (0.8 - 1.0) | 1.1 (1.0 - 1.2) |
| **2003** | 0.9 (0.8 - 1.0) | 1.1 (1.0 - 1.2) | 0.9 (0.8 - 1.0) | 1.0 (0.9 - 1.1) |
| **2004** | 0.9 (0.8 - 0.9) | 1.0 (0.9 - 1.1) | 0.9 (0.9 - 1.0) | 0.9 (0.8 - 1.0) |
| **2005** | 0.9 (0.8 - 1.0) | 0.8 (0.8 - 0.9) | 1.0 (0.9 - 1.0) | 0.9 (0.8 - 1.0) |
| **2006** | 0.9 (0.8 - 0.9) | 0.9 (0.8 - 0.9) | 0.9 (0.8 - 1.0) | 0.9 (0.9 - 1.0) |
| **2007** | 0.9 (0.8 - 1.0) | 0.9 (0.8 - 0.9) | 0.8 (0.8 - 0.9) | 1.0 (0.9 - 1.1) |
| **2008** | 0.9 (0.8 - 1.0) | 0.9 (0.8 - 1.0) | 0.9 (0.8 - 0.9) | 1.0 (0.9 - 1.1) |
| **2009** | 0.9 (0.8 - 0.9) | 1.0 (0.9 - 1.1) | 0.9 (0.8 - 1.0) | 1.1 (1.0 - 1.2) |
| **2010** | 1.0 (0.9 - 1.1) | 1.0 (0.9 - 1.1) | 0.9 (0.8 - 1.0) | 1.1 (1.0 - 1.2) |
| **2011** | 1.1 (1.0 - 1.3) | 1.0 (0.9 - 1.1) | 1.1 (1.0 - 1.2) | 1.1 (1.0 - 1.2) |
| **2012** | 1.1 (1.0 - 1.2) | 1.2 (1.1 - 1.3) | 1.1 (1.1 - 1.2) | 1.2 (1.1 - 1.3) |
| **2013** | 1.2 (1.1 - 1.3) | 1.2 (1.1 - 1.3) | 1.3 (1.2 - 1.4) | 1.4 (1.3 - 1.5) |
| **2014** | 1.5 (1.4 - 1.6) | 1.5 (1.4 - 1.6) | 1.5 (1.4 - 1.6) | 1.5 (1.4 - 1.6) |
| **2015** | 1.7 (1.5 - 1.8) | 1.6 (1.5 - 1.7) | 1.9 (1.8 - 2.0) | 1.8 (1.7 - 1.9) |
| **2016** | 1.9 (1.8 - 2.1) | 1.8 (1.7 - 1.9) | 2.2 (2.1 - 2.3) | 2.2 (2.0 - 2.3) |
| **2017** | 2.1 (2.0 - 2.3) | 2.0 (1.9 - 2.1) | 2.5 (2.4 - 2.6) | 2.4 (2.3 - 2.5) |
| **2018** | 2.5 (2.3 - 2.6) | 2.3 (2.2 - 2.4) | 3.0 (2.9 - 3.1) | 2.8 (2.7 - 2.9) |
| **2019** | 2.6 (2.5 - 2.8) | 2.6 (2.5 - 2.8) | 3.3 (3.2 - 3.4) | 3.3 (3.1 - 3.4) |
| **2020** | 2.9 (2.7 - 3.1) | 3.1 (3.0 - 3.3) | 3.6 (3.4 - 3.7) | 3.6 (3.4 - 3.7) |
| **2021** | 3.5 (3.4 - 3.7) | 3.7 (3.6 - 3.9) | 4.2 (4.1 - 4.4) | 4.5 (4.4 - 4.7) |
| **2022** | 3.9 (3.7 - 4.1) | 3.9 (3.7 - 4.0) | 4.5 (4.3 - 4.6) | 4.8 (4.7 - 5.0) |
| **2023** | 4.3 (4.1 - 4.5) | 4.1 (4.0 - 4.3) | 4.7 (4.6 - 4.8) | 5.2 (5.0 - 5.4) |
| **Total** | **1.7 (1.6 - 1.8)** | **1.7 (1.6 - 1.8)** | **1.9 (1.8 - 1.9)** | **1.9 (1.8 - 2.0)** |

**Supplemental Table 8: Cardiogenic Shock-related Age-Adjusted Mortality Rates per 100,000, Stratified by Urban-Rural Classification, in Adults with Heart Failure in the United States, 1999 to 2020.**

| **Age-Adjusted Rate (95% CI)** | | |  |
| --- | --- | --- | --- |
| **Year** | **Urban** | **Rural** |  |
| **1999** | 1.2 (1.1 - 1.2) | 1.5 (1.4 - 1.7) |  |
| **2000** | 1.1 (1.0 - 1.1) | 1.4 (1.3 - 1.5) |  |
| **2001** | 1.0 (0.9 - 1.0) | 1.3 (1.1 - 1.4) |  |
| **2002** | 1.0 (0.9 - 1.0) | 1.1 (1.0 - 1.2) |  |
| **2003** | 1.0 (0.9 - 1.0) | 1.0 (0.9 - 1.1) |  |
| **2004** | 0.9 (0.9 - 1.0) | 1.1 (1.0 - 1.2) |  |
| **2005** | 0.9 (0.9 - 1.0) | 1.1 (1.0 - 1.2) |  |
| **2006** | 0.9 (0.8 - 0.9) | 0.9 (0.8 - 1.0) |  |
| **2007** | 0.9 (0.8 - 0.9) | 1.0 (0.9 - 1.1) |  |
| **2008** | 0.9 (0.9 - 0.9) | 1.0 (0.9 - 1.1) |  |
| **2009** | 1.0 (0.9 - 1.0) | 1.0 (0.9 - 1.1) |  |
| **2010** | 0.9 (0.9 - 1.0) | 1.1 (1.0 - 1.2) |  |
| **2011** | 1.1 (1.0 - 1.1) | 1.3 (1.1 - 1.4) |  |
| **2012** | 1.1 (1.0 - 1.2) | 1.4 (1.3 - 1.5) |  |
| **2013** | 1.2 (1.2 - 1.3) | 1.4 (1.3 - 1.5) |  |
| **2014** | 1.5 (1.4 - 1.5) | 1.7 (1.6 - 1.8) |  |
| **2015** | 1.7 (1.7 - 1.8) | 2.0 (1.8 - 2.1) |  |
| **2016** | 2.0 (1.9 - 2.0) | 2.4 (2.2 - 2.5) |  |
| **2017** | 2.3 (2.2 - 2.3) | 2.6 (2.5 - 2.8) |  |
| **2018** | 2.6 (2.6 - 2.7) | 3.1 (2.9 - 3.2) |  |
| **2019** | 3.0 (2.9 - 3.0) | 3.5 (3.3 - 3.6) |  |
| **2020** | 3.2 (3.2 - 3.3) | 3.9 (3.7 - 4.1) |  |
| **Total** | **1.5 (1.5 - 1.5)** | **1.7 (1.7 - 1.8)** |  |
| The data for urbanization is only available till 2020 in the CDC Wonder Database. | | |  |
|  |  |  |  |
